# Error in Article Information Section

**DOI:** 10.1001/jamanetworkopen.2020.33114

**Published:** 2020-12-15

In the Original Investigation titled “Evaluation of the Use of Combined Artificial Intelligence and Pathologist Assessment to Review and Grade Prostate Biopsies,”^1^ published November 12, 2020, the author affiliations for Martin C. Stumpe, PhD, were incorrectly listed in the Article Information section. Dr Stumpe’s surname should have appeared between those of authors Tan and Jiang indicating affiliation with Google Health, Palo Alto, California, at the time of author contributions; the last line of the Author Affilations paragraph should have appeared as “Now with Tempus Labs, Chicago, Illinois (Stumpe).” This article has been corrected.^1^
